# A Comprehensive Study on the Volatile Flavor Profile and Microbial Community of Stir-Fried Sour Shrimp Paste

**DOI:** 10.3390/foods15132338

**Published:** 2026-07-01

**Authors:** Jiahui Shi, Weixi Yang, Huangqing Yang, Yifei Li, Kangli Guo, Wenlu Li, Yanbo Wang, Yuxiang Gu

**Affiliations:** 1 School of Food and Health, Beijing Technology and Business University, Beijing 100048, China; s3314454093@163.com (J.S.); vichy_yangweixi@163.com (W.Y.); 17803291818@163.com (H.Y.); 18833246063@163.com (Y.L.); guokl@btbu.edu.cn (K.G.); liwenlu13@163.com (W.L.); 2 Key Laboratory of Geriatric Nutrition and Health, Ministry of Education, Beijing Technology and Business University, Beijing 100048, China; 3 NHC Specialty Laboratory of Food Safety Risk Assessment and Standard Development, Beijing Technology and Business University, Beijing 100048, China

**Keywords:** traditional fermented food, aroma compounds, quantitative descriptive analysis, high-throughput sequencing, HS-SPME-GC-MS, bacterial and fungal diversity

## Abstract

Stir-frying is an important process for terminating fermentation and improving the flavor of sour shrimp paste. This study investigated the flavor characteristics, volatile profiles, and post-storage microbial community of stir-fried sour shrimp paste. Sensory evaluation demonstrated that it exhibits a well-balanced flavor profile, characterized by fruity, soy sauce, spicy, and salty notes. Based on HS-SPME-GC-MS analysis, a total of 68 volatile compounds were identified, of which 26 made notable contributions to the aroma. Among them, esters were the most diverse group, with ethyl butyrate, ethyl acetate, and ethyl 2-methylbutyrate serving as the main contributors to the fruity aroma. Acids such as butanoic acid, acetic acid, and 2-methylbutanoic acid far exceeded their thresholds, linking to sour and spicy notes. Microbial community analysis revealed that low-abundance fermentative bacteria, such as *Vagococcus* and *Levilactobacillus*, remained detectable after storage, whereas *Pseudomonas* and *Acinetobacter* were identified as the most probable spoilage organisms. These findings provide a systematic understanding of the unique flavor of stir-fried sour shrimp paste and its potential microbial risks during storage, providing a basis for the application of stir-frying technology.

## 1. Introduction

As a traditional intangible cultural heritage delicacy originating from southwestern China, sour shrimp paste traces its history back over 400 years and is highly appreciated by consumers for its distinctive flavor. Its traditional production method typically employs shrimp as the primary ingredient, which then undergoes prolonged co-fermentation with cereals and spices to form a characteristic acidic fermentation system [1]. This fermentation model is also prevalent in other traditional Asian fermented foods, such as *suanyu* and *suanrou*, sharing similar features in both process characteristics and microbial succession [2,3]. Existing studies have mainly focused on the relationships among the physicochemical properties, flavor characteristics, and microbial community structure of raw sour shrimp paste [1,4]. However, in traditional consumption practices, sour shrimp paste is usually stir-fried before consumption to fully release its characteristic aroma and flavor.

From an industrial perspective, as a fermented product, raw sour shrimp paste continues to undergo fermentation during storage and distribution. This ongoing fermentation may lead to time-dependent changes in flavor and quality, resulting in reduced product stability and thereby limiting its large-scale commercialization. From the perspective of consumer convenience, stir-fried sour shrimp paste (SFSP) eliminates the time-consuming and experience-dependent cooking step required before consumption, allowing the product to be consumed directly while facilitating better control of its overall flavor quality. Similar to *suanyu*, cooked products are generally more preferred by consumers [5]. Therefore, to improve product stability and meet industrial demands, stir-frying has gradually been introduced into the processing of sour shrimp paste. Meanwhile, to satisfy the growing demand for ready-to-eat products, SFSP has been developed, providing a more convenient product form with a standardized flavor profile for broader culinary applications.

In addition, high-temperature stir-frying terminates fermentation by inactivating fermentative microorganisms while simultaneously inhibiting spoilage and pathogenic microorganisms. By fixing microbial metabolic activity and flavor at a specific stage, this process prevents further microbial-induced changes, thereby improving product stability and controllability. Moreover, heat treatment can alleviate undesirable odors and quality deterioration commonly observed in fermented aquatic products by inactivating endogenous enzymes, thereby slowing excessive proteolysis and the further accumulation of biogenic amines [6]. Notably, thermal processing can significantly affect the flavor of food [7]. For example, it can promote the formation and release of volatile compounds in soy sauce, enhancing aroma intensity, while high-temperature cooking may also lead to the loss of certain aromatic components [8,9]. Therefore, stir-frying not only terminates fermentation but also contributes to flavor stabilization and improved microbiological safety.

Consequently, elucidating the flavor characteristics and microbial community structure of SFSP is of considerable importance. Based on the above considerations, this study focuses on SFSP as the research object and systematically analyzes its overall flavor profile and post-storage microbial composition, integrating sensory evaluation, volatile flavor compound analysis, and high-throughput sequencing techniques. The research results aim to elucidate the flavor characteristics and key flavor compounds of SFSP, and to assess its biological stability and microbiological safety risks. The findings will support the scientific quality control and safety assurance of the product. Additionally, this study enriches the understanding of flavor chemistry in fermented foods from the perspective of cooked products, providing a scientific basis for the industrial processing and quality control of traditional fermented foods.

## 2. Materials and Methods

### 2.1. Reagents and Chemicals

C_6_–C_24_ n-alkanes were obtained from Sigma-Aldrich (St. Louis, MO, USA). Sodium chloride of analytical grade was supplied by Beijing Chemical Works (Beijing, China). Boric acid, magnesium oxide, methyl red, and bromocresol green were purchased from Macklin Biochemical Co., Ltd. (Shanghai, China). Hydrochloric acid standard titration solution was supplied by Yida (Quanzhou, China) Technology Co., Ltd. (Fujian, China). An ethanol solution of analytical grade was used.

### 2.2. Samples

**Figure 1:**
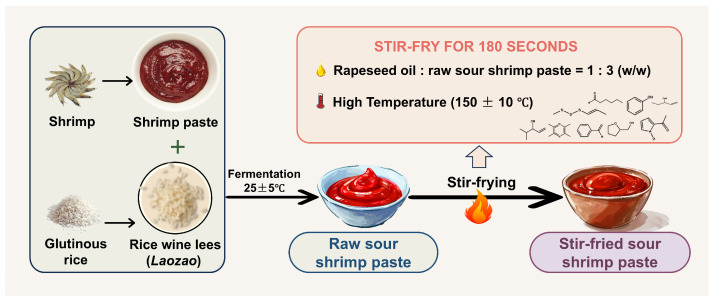
Schematic illustration of preparation of SFSP.

Five SFSP samples, designated as SP1–SP5, were collected from the market in Guizhou Province, China. The samples were prepared by stir-frying raw sour shrimp paste obtained from different manufacturers. Briefly, the primary shrimp paste fermentation involved mixing fresh shrimp (*Acetes chinensis*) with 10% (*w*/*w*) sodium chloride and fermenting the mixture for 12 months. Concurrently, fermented glutinous rice (*Laozao*) was produced by steaming soaked glutinous rice (*Oryza sativa* L. var. *glutinosa*), and inoculating it with 0.2% (*w*/*w*) sweet *qu* (traditional starter contains *Rhizopus oryzae* and *Saccharomyces cerevisiae*). The inoculated rice was then fermented at 25 ± 2 °C for 3 days. For the secondary fermentation, the primary fermented shrimp paste, *Laozao*, and chili powder were blended at a mass ratio of 6:3:1 and fermented at 25 ± 5 °C. Finally, the fermented sour shrimp paste was stir-fried using rapeseed oil at a ratio of rapeseed oil to raw sour shrimp paste of 1:3 (*w*/*w*). The mixture was heated at 150 ± 10 °C under continuous stirring for 180 s to obtain the final SFSP product. After sampling, the SFSP samples were immediately subjected to pH determination, total volatile basic nitrogen (TVB-N) analysis, sensory evaluation, and volatile flavor analysis to characterize the physicochemical and flavor properties of the products after stir-frying. To further evaluate the safety of SFSP during storage, the remaining samples were sealed in sterile glass jars and stored at room temperature for one month before microbial community analysis. The preparation process of SFSP is illustrated in Figure 1.

### 2.3. Analysis of pH and TVB-N

The pH value was measured according to the method described by Lv et al. [10], using a calibrated digital pH meter after sample homogenization. Each sample was analyzed in duplicate. TVB-N was determined using an automatic Kjeldahl nitrogen analyzer according to the Chinese national standard method (GB 5009.228–2016) [11]. Briefly, approximately 10 g of sample (accurate to 0.001 g) was weighed into a distillation tube, followed by the addition of 75 mL of distilled water. The mixture was thoroughly shaken to ensure uniform dispersion and allow for extraction for 30 min. The instrument was then operated according to the manufacturer’s instructions, and a reagent blank was measured after the system reached stable operating conditions. Subsequently, 1 g of magnesium oxide was added to the prepared sample in the distillation tube, which was immediately connected to the distillation unit, and the determination was carried out under the preset instrument conditions. Each sample was analyzed in triplicate.

### 2.4. Sensory Evaluation

The evaluation was conducted by a sensory panel consisting of 20 trained members (9 males and 11 females, aged 20–35 years). All participants were informed that they were completely voluntary and free to participate in these subjects. Ethical approval for the involvement of human subjects in this study was granted by the Scientific Research Ethics Committee of Beijing Technology and Business University (reference number 2025–127).

All of the sensory analyses were performed at controlled room temperature (20 °C). Samples, each accurately weighed at 3.0 g, were placed in 50 mL opaque vials and coded with a three-digit random number. The samples were presented simultaneously to the panels to evaluate the aroma. Each sample was evaluated independently by all 20 trained panelists, and the sensory scores were expressed as the mean value obtained from the 20 evaluations. Seven aroma attributes, including fishy, salty, stink, sour, fruity, spicy, and soy sauce, were assessed using an 11-point scale, where “0” indicated no perceived intensity and “10” represented extremely strong intensity. Corresponding reference standards, including commercially available odorants and natural products, were used to define each of these attributes (Table 1).

### 2.5. Analysis of Volatile Compounds

#### 2.5.1. Headspace Solid-Phase Microextraction–Gas Chromatography–Mass Spectrometry (HS-SPME-GC-MS) Analysis

The sample (3.00 g) was accurately weighed into a 20 mL headspace vial, followed by the addition of 3 mL of saturated NaCl solution and 10 μL of internal standard (2-methyl-3-heptanone). The vial was immediately sealed with a PTFE–silicone septum. The mixture was equilibrated at 40 °C for 30 min under agitation at 250 rpm. Subsequently, a DVB/CAR/PDMS fiber was exposed to the headspace for extraction for 30 min under the same conditions. After extraction, the fiber was inserted into the GC-MS injection port and thermally desorbed at 250 °C for 8 min. HS-SPME-GC-MS analysis was performed in duplicate for each sample, and the results are presented as the mean values of two independent determinations.

GC conditions: Separation was performed on an HP-INNOWAX capillary column (60 m × 0.25 mm × 0.25 μm). The inlet temperature was set at 250 °C, and the analysis was conducted in split mode with a split ratio of 10:1. High-purity helium was used as the carrier gas at a constant flow rate of 1.0 mL/min. The oven temperature program was as follows: initial temperature of 40 °C held for 5 min, increased to 250 °C at a rate of 5 °C/min, and held at 250 °C for 5 min.

Mass spectrometry conditions: the mass spectrometer was operated in electron ionization (EI) mode at 70 eV. The ion source temperature was set at 230 °C, and the quadrupole temperature was maintained at 150 °C. Mass spectra were acquired in full-scan mode over an *m*/*z* range of 30–350.

#### 2.5.2. Qualitative and Quantitative Analysis of Volatile Compounds

Compounds were tentatively identified using the NIST mass spectral library and retention indices (RI) calculated based on a series of n-alkanes (C_6_–C_24_) analyzed under identical chromatographic conditions. RI values were calculated according to the following formula:

(1) $${RI}_{x}=100n+\frac{t_{x}-t_{n}}{t_{n+1}-t_{n}}$$

where RI_x_ is the retention index of the volatile compound; n represents the carbon number of the n-alkane eluting immediately before the compound, while t_x_, t_n_, and t_n+1_ correspond to the retention times of the target compound, the preceding n-alkane (C_n_), and the following n-alkane (C_n+1_), respectively.

Volatile compounds were semi-quantified using an internal standard method, and the relative concentration of each compound was calculated according to the following formula:

(2) $$C_{x}=\frac{A_{x}\times C_{0}}{A_{0}\times m_{1}}$$

where C_x_ is the relative concentration of the target volatile compound, μg/kg; A_x_ and A_0_ are the peak areas of the target volatile compound and the internal standard, respectively; C_0_ is the concentration of the internal standard, μg/kg; m_1_ is the sample mass, g.

### 2.6. Analysis of Microbial Community

Total microbial genomic DNA was isolated from the samples using an E.Z.N.A.^®^ Soil DNA Kit (Omega Bio-tek, Norcross, GA, USA) following the manufacturer’s protocol. DNA quality and concentration were evaluated by 1.0% agarose gel electrophoresis and a NanoDrop 2000 spectrophotometer (Thermo Scientific, Waltham, MA, USA), and qualified DNA was stored at −80 °C until further analysis. For each sample, DNA extraction and subsequent microbial community analyses were conducted in triplicate.

The V3–V4 hypervariable region of the bacterial 16S rRNA gene was amplified using primers 338F (5′-ACTCCTACGGGAGGCAGCAG-3′) and 806R (5′-GGACTACHVGGGTWTCTAAT-3′), while the fungal ITS region (ITS1/ITS2) was amplified using primers ITS1F (5′-CTTGGTCATTTAGAGGAAGTAA-3′) and ITS2R (5′-GCTGCGTTCTTCATCGATGC-3′). PCR amplification was performed on a T100 thermal cycler (BIO-RAD, Hercules, CA, USA) under the following conditions: initial denaturation at 95 °C for 3 min, followed by 27 cycles of denaturation at 95 °C for 30 s, annealing at 55 °C for 30 s, extension at 72 °C for 45 s, and a final extension at 72 °C for 10 min.

PCR products were recovered from 2% agarose gels, purified using a PCR Clean-Up Kit (YuHua, Shanghai, China), and quantified with a Quantus™ fluorometer (Promega, Madison, WI, USA). Purified amplicons were pooled in equimolar concentrations and sequenced using an Illumina MiSeq PE300 or NovaSeq PE250 platform (Illumina, San Diego, CA, USA) by Majorbio Bio-Pharm Technology Co., Ltd. (Shanghai, China). Raw sequencing data were demultiplexed, quality-filtered using Fastp (v0.20.0), and merged with FLASH (v1.2.7). Operational taxonomic units (OTUs) were clustered at 97% sequence similarity using UPARSE (v7.1), and chimeric sequences were removed. Taxonomic classification of representative OTU sequences was conducted using the RDP Classifier (v2.2) against the Silva 16S rRNA database (v138) with a confidence threshold of 0.7. Sequencing data were deposited in the NCBI Sequence Read Archive (SRA) under BioProject accession number PRJNA1277469.

### 2.7. Statistical Analysis

Statistical analyses were performed using IBM SPSS Statistics 27. Differences among treatments were analyzed by one-way analysis of variance (ANOVA) followed by Duncan’s multiple range test, and statistical significance was determined at *p* < 0.05. Pearson correlation analysis was used to evaluate the relationships among variables. The resulting correlation matrix was visualized as a heatmap using the ChiPlot platform (https://www.chiplot.online/). Bioinformatic analyses of microbial communities were performed on the Majorbio Cloud platform (https://cloud.majorbio.com).

## 3. Results and Discussion

### 3.1. pH and TVB-N of SFSP Samples

The pH value reflects the overall acidity of the sample after stir-frying processes (Table 2). For the SFSP samples, pH values ranged from 3.76 to 7.28. Similar pH ranges have been reported for other acidic fermented products, such as *suanyu*, in which pH values are generally maintained between 4.00 and 7.00 [12], indicating that an acidic environment is a common characteristic contributing to flavor development and product stability. Compared with pH values reported for raw sour shrimp paste in previous studies [1,4], SFSP samples exhibited an overall higher pH level, suggesting that the stir-frying process can mitigate the inherent acidity. This effect has also been observed in other similar fermented products. For example, previous studies have shown that the pH of *suanrou* increased following different frying treatments [13]. The pH value of fermented foods correlates with the accumulation of organic acids, primarily lactic acid and acetic acid, produced during the fermentation process [14,15]. Notably, these organic acids are widely present in acidic fermented foods such as *suanyu*, *suanrou*, and sour shrimp paste [1,5,16]. In addition, the contents of lactic acid and acetic acid may decrease after stir-frying [5]. This reduction in acidity may be associated with the volatilization or evaporation of organic acids during heating. Meanwhile, this decrease acidity may be attributed to the thermal degradation of organic acids under high temperatures or to decarboxylation reactions occurring during heating [17,18].

TVB-N is widely used as an indicator of spoilage in aquatic products and as a marker for the degree of fermentation in fermented foods [19]. The TVB-N content of samples varied from 13.21 to 96.80 mg/100 g, remaining well below commonly referenced spoilage thresholds (450 mg/100 g, SB/T 10525 2009) [20], indicating sound product quality. There are significant differences in TVB-N content among different SFSP samples. Previously reported TVB-N levels in *suanyu* also showed considerable variation, ranging from 9.41 to 124.67 mg/100 g [12]. In fermented foods, the formation of TVB-N mainly originates from the degradation of proteins, leading to the accumulation of alkaline nitrogenous compounds such as ammonia and various amines [21]. Higher TVB-N values indicate more extensive protein breakdown and greater production of amines and other alkaline substances [22]. Variations in TVB-N levels are commonly associated with specific spoilage microorganisms, endogenous enzymatic activity, and protein-hydrolyzing bacteria [22,23,24]. In addition, the fermentation degree of raw sour shrimp paste may affect the TVB-N level in SFSP.

### 3.2. Sensory Characteristics of SFSP

**Figure 2:**
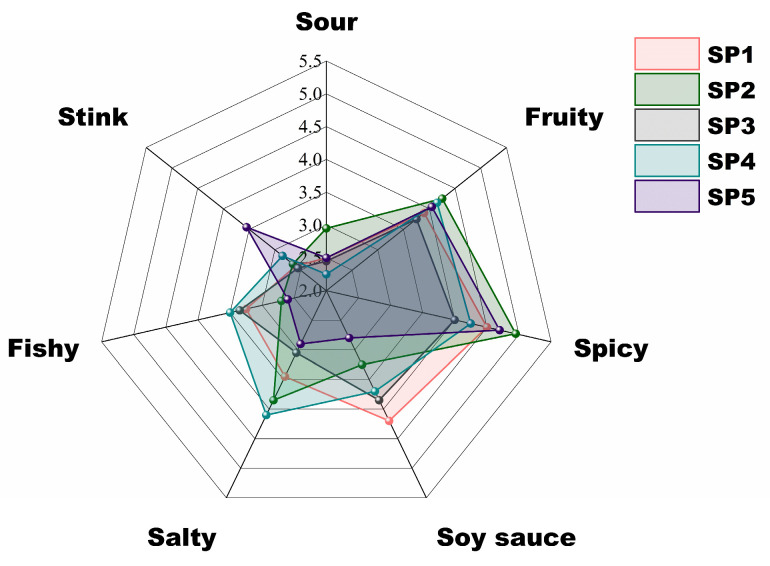
Sensory characteristics of SFSP samples.

Based on the sensory evaluation results, a radar chart of the aroma profile for SFSP was plotted according to the scores of each flavor attribute (Figure 2). Statistical comparisons of the sensory data were conducted, and the detailed results are provided in Supplementary Table S1. The statistical analysis revealed that there were no significant differences among the five SFSP samples in most sensory attributes, including spicy, fruity, sour, salty, fishy, and stinky attributes, indicating a generally consistent sensory profile across samples. Only the soy sauce attribute showed significant variation among samples, suggesting that this characteristic may be more sensitive to differences in raw materials or fermentation conditions. Overall, SFSP exhibited a characteristic aroma profile dominated by fruity, soy sauce, spicy, and salty attributes, whereas sour, fishy, and stinky notes showed relatively low intensities, indicating a balanced and generally acceptable sensory quality [25]. Specifically, the intensity of fruity aroma in SFSP ranged from 3.75 to 4.25, while the intensity of spicy aroma was approximately 4.00–4.95. These attributes showed relatively concentrated scores across different samples, suggesting that fruity and spicy aromas are stable and consistent sensory characteristics shared among SFSP samples. A fruity-dominated aroma profile has also been widely reported in traditional fermented products produced using similar processing methods, such as *suanyu* [26]. In contrast, soy sauce and salty aromas exhibited greater variability among different samples, with intensity ranges of 2.8–4.2 and 2.9–4.1, respectively. Specifically, SP1 and SP2 exhibit the most pronounced soy sauce and fruity aroma characteristics, which align well with their lower pH values and higher TVB-N content. Higher TVB-N levels are generally considered to reflect more extensive protein degradation and a more advanced stage of fermentation [27]. Moreover, previous studies have suggested that fruity and soy sauce aroma characteristics tend to be associated with higher fermentation intensity [27,28]. In contrast, SP5 has the lowest TVB-N content, indicating a relatively weaker fermentation degree and a less distinctive overall flavor profile, particularly with weaker soy sauce and fishy notes. Meanwhile, SP4 has a relatively higher pH, which may reflect limited acid production during the second stage of fermentation, resulting in overall sensory characteristics more similar to traditional shrimp paste, dominated by fishy and salty flavors, with higher pH and weaker sour attribute [29].

### 3.3. Composition of Volatile Compounds

**Figure 3:**
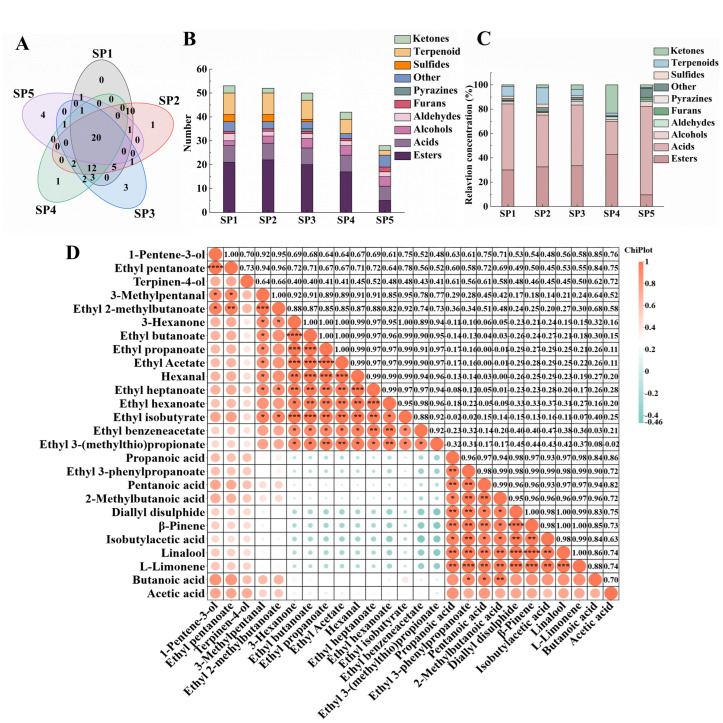
HS-SPME-GC-MS analysis of aroma compounds in SFSP. Venn diagram (**A**); number of volatile compounds (**B**); relative contribution of volatile compounds (**C**); Pearson correlation heatmap of 26 aroma compounds (**D**). *: *p* < 0.05; **: *p* < 0.01; ***: *p* < 0.001; ****: *p* < 0.0001.

The volatile compounds in SFSP were analyzed using HS-SPME-GC-MS. As shown in Table 3, a total of 68 volatile compounds were identified in the samples, including esters (28), terpenes (9), acids (7), alcohols (5), and other compounds. The Venn diagram clearly illustrates the distribution and shared volatile compounds among the samples (Figure 3A). All 20 volatile compounds shared by the samples can be regarded as the stable flavor compounds of the aroma composition of SFSP, mainly including 5 esters, 2 alcohols, 6 acids, and 7 other compounds. As shown in Figure 3B,C, a diverse range of volatile compounds was detected across all samples. SP1 exhibited the highest diversity of flavor compounds, while SP5 contained the lowest. Notably, SP5 also exhibited the lowest TVB-N content among all samples (Table 2), which is a key indicator reflecting the degree of protein and amino acid degradation during fermentation, with its content generally increasing as the fermentation process proceeds [30]. Furthermore, a common characteristic was observed in the compositional profiles of all samples: esters and acids were the most abundant compound classes, both in terms of number and concentration.

#### 3.3.1. Esters

**Figure 4:**
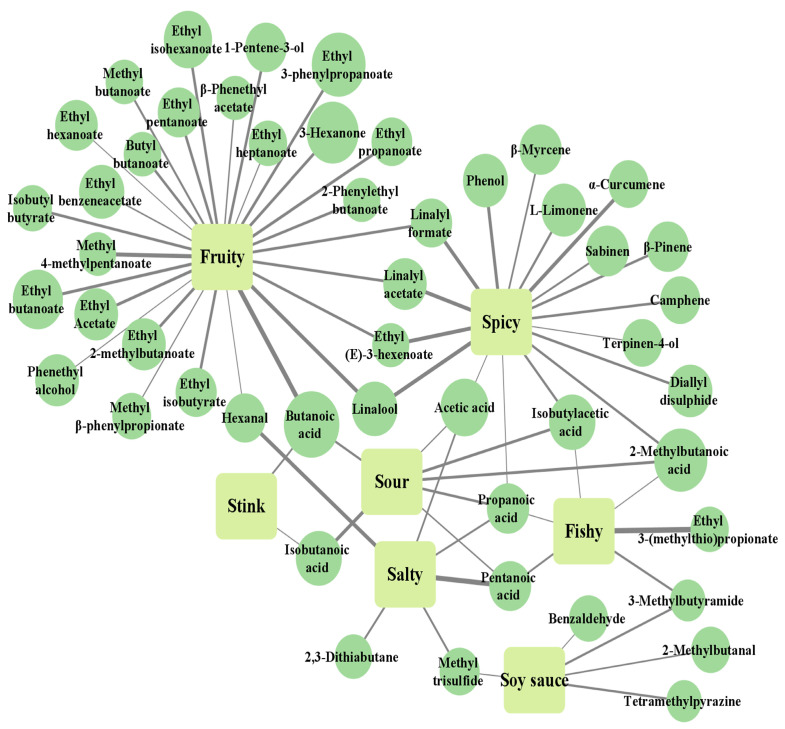
Relationship between aroma compounds and sensory characteristics of SFSP.

Esters were identified as the major aroma-active compounds in SFSP, with total concentrations ranging from 11.48 to 412.94 μg/g, accounting for 9.58–42.86% of the total volatile components (Figure 3C). Notably, esters generally exhibit low odor thresholds [40]. Esters in SFSP were dominated by ethyl esters, most of which were fatty acid ethyl esters. These compounds were present at relatively high concentrations and exhibited low odor thresholds. For example, ethyl acetate was detected at 9.75 μg/g, with a threshold of 5 μg/kg, ethyl 2-methylbutanoate at 7.27 μg/g with a threshold of 1 μg/kg, and ethyl 3-methylbutanoate at 3.16 μg/g with a threshold of 3 μg/kg, all exceeding their respective odor thresholds. These esters are associated with typical fruity notes such as pineapple, banana, strawberry, cherry, and green apple [41]. Collectively, these high-abundance, low-threshold esters played a crucial role in shaping the fruity sensory attribute of SFSP (Figure 4) and constituted the chemical basis of its characteristic fruity aroma.

Among the five esters shared by all SFSP samples, three exhibited both relatively high concentrations and low odor thresholds, namely ethyl 3-phenylpropanoate, ethyl butanoate, and ethyl 2-methylbutanoate. Among them, ethyl 3-phenylpropanoate was the most abundant ester, with concentrations ranging from 2.64 to 174.64 μg/g, which were markedly higher than its odor threshold (1.6 μg/kg), imparting pronounced fruity, rose, honey, and rum aroma notes. Ethyl butanoate was detected at concentrations of 1.59–188.21 μg/g, far exceeding its odor threshold (20 μg/kg), and is known to contribute characteristic banana and pineapple aromas. In addition, ethyl benzeneacetate was present at concentrations of 3.00–34.67 μg/g, also substantially higher than its reported odor threshold (575 μg/kg), further reinforcing the fruity sensory attribute.

Rich ester profiles have also been reported in other traditionally fermented meat products produced using similar processing methods. For instance, 44 esters were identified in sour shrimp paste and *suanrou* [1,3], whereas 13 esters were detected in *suanyu* [5]. Although esters predominated in the volatile profiles of all these products, the composition of high-abundance esters differed among them. Similar to the present study, ethyl acetate and ethyl 3-methylbutanoate were also present at high levels in *suanyu*, whereas ethyl lactate exhibited higher abundance in *suanyu* [26], which may be attributed to differences in raw materials and fermentation pathways. Notably, multiple ethyl esters, including ethyl 3-phenylpropanoate and ethyl isohexanoate, were already detected at relatively high levels during the sour shrimp paste stage. These esters are likely derived from microbial alcoholic fermentation during glutinous rice wine production [42]. The abundance of ethyl esters is known to enhance the pleasant aroma while reducing undesirable odors [43,44].

#### 3.3.2. Acids

Acids play an important role in the formation of esters and aldehydes. In this study, seven acid compounds were detected in the samples. Butanoic acid was present at a concentration of 108.50 μg/g, which was significantly higher than its sensory threshold of 2.5 μg/g, and mainly imparted cheesy and rancid odors [45], potentially associated with the stink sensory attribute. Pentanoic acid was detected at a concentration of 3.55 μg/g; although its content was relatively low, its sensory threshold is extremely low at only 0.037 μg/kg, and it is characterized by strong putrid, rancid, sickening, acidic, and sweaty odors, suggesting that it may exert a pronounced influence on the overall aroma even at low concentrations. Acetic acid reached 33.34 μg/g, markedly exceeding its threshold (22 μg/g), and contributed typical sour and vinegar, serving as the contributor to acidity perception in SFSP (Figure 4). Likewise, the concentration of 2-methylbutanoic acid and propanoic acid was far above their thresholds and exhibited acidic and roquefort cheese-like aromas, which may be closely related to the sensory attributes of sour, spicy, and mild fishy note observed in the sensory evaluation (Figure 4). Isobutanoic acid, with a concentration of 16.55 μg/g, exhibited characteristic sour, cheesy, dairy, and putrid aromas, thereby reinforcing the fermented flavor characteristics of the product.

From a fermentation perspective, several of these acids, particularly acetic acid, have also been reported in sour shrimp paste and other acidic fermented aquatic products such as *suanyu* [1,26], indicating that short-chain fatty acids are common metabolic products in such fermentation systems. The accumulation of these organic acids contributes to the formation of a low-pH environment, which effectively inhibits the growth of spoilage microorganisms [46].

#### 3.3.3. Alcohols

A total of five alcohols were identified in the samples, including isobutanol, 1-butanol, 1-pentene-3-ol, 2-methyl-4-butanol, and phenethyl alcohol. The detected alcohols generally exhibited relatively high odor thresholds and therefore contributed a limited direct impact to the overall aroma. Notably, only 1-pentene-3-ol was detected at a concentration exceeding its odor threshold, imparting distinct horseradish-like, green, radish, chrysanthemum, vegetable, and fruity notes. In addition, phenethyl alcohol, a commonly reported alcohol in fermented fish products such as *suanyu* and *dushan* sour shrimp paste, is characterized by typical rose and honey-like aromas and is considered an important contributor to the aroma profile of *dushan* sour shrimp paste [4,47]. Alcohols are generally regarded as by-products of lipid oxidation, primarily derived from the oxidative degradation of polyunsaturated fatty acids [48]. Their formation reflects the role of lipid transformation during the fermentation and stir-frying process in shaping the overall flavor of SFSP. However, in traditional fermented aquatic products such as *suanyu*, the formation of alcohols is not solely attributable to lipid transformation but is also closely associated with microbial metabolic activities during fermentation [47].

#### 3.3.4. Terpenes

In this study, a total of nine terpenoid compounds were detected in the samples, most of which exhibited relatively low odor thresholds. β-Pinene was present at a concentration of 8.22 μg/g, far exceeding its odor threshold of 0.14 μg/g, and contributed characteristic dry, woody, resinous, pine, and green aromas. L-Limonene was detected at 8.79 μg/g, significantly higher than its threshold of 38 μg/kg, imparting typical terpene, pine, herbal, and peppery notes. Linalool showed a relatively high concentration of 15.90 μg/g, well above its threshold of 15 μg/kg, and is characterized by citrus, floral, sweet, woody, and rose aromas, making it one of the most aroma-active terpenes in the samples. In addition, terpinen-4-ol was detected at 1.27 μg/g, exceeding its threshold of 0.11 μg/g, and contributed peppery, woody, and mildly sweet notes.

These high-abundance, low-threshold terpenes are likely derived from the addition of spice ingredients, such as chili peppers and other plant-based seasonings. Previous studies have identified linalool as a key aroma-active compound in fermented chili products [49], and its high level in SFSP further highlights the important contribution of spices to flavor development. Consistent with sensory evaluation results (Figure 4), these terpenes showed a strong association with the spicy sensory attribute and constitute an important chemical basis for the characteristic spicy aroma and flavor distinctiveness of SFSP. Moreover, terpenoid compounds are commonly reported in stir-fried seasoning pastes and cooked sauce products, suggesting that their presence may result not only from the direct incorporation of spices but also from thermal release and transformation processes during cooking [50].

#### 3.3.5. Other Compounds

Aldehydes and ketones are mainly generated through lipid oxidation and amino acid degradation and generally exhibit low sensory thresholds. In this study, six aldehyde and ketone compounds were identified in the SFSP samples, among which 3-hexanone, hexanal, and 3-methylpentanal were present at relatively high concentrations, contributing sweet and fruity aroma notes [51]. In addition to aldehydes and ketones, sulfides and pyrazines also play a critical role in the flavor formation of SFSP. The concentrations of sulfides detected were markedly higher than their respective sensory thresholds. Specifically, 2,3-dithiabutane was detected at 0.55 μg/g, far exceeding its threshold of 2.2 μg/kg; methyl trisulfide was present at 2.10 μg/g, above its threshold of 4 μg/kg; and diallyl disulphide reached 13.44 μg/g, substantially higher than its threshold of 0.22 μg/kg. These sulfides typically impart onion, cooked, and savory aroma characteristics. Pyrazine compounds, as lipid oxidation-derived products involved in Maillard reactions, are known to exhibit nutty, musty, chocolate, coffee, cocoa, lard, and burnt aroma notes. Owing to their low sensory thresholds, sulfides and pyrazines together constitute the primary contributors to the characteristic soy sauce aroma of SFSP.

Furthermore, minor amounts of heterocyclic and phenolic compounds, including indole and phenol, were also detected in SFSP. Indole is mainly derived from the microbial degradation of tryptophan and may produce putrid or musty odors at elevated concentrations [52]. Phenol, characterized by plastic-like and rubber-like odors, has been reported as a key aroma compound in *Wangzhihe* stinky tofu and its brine [53]. The presence of these compounds may be associated with the off-odor attributes observed in sensory evaluation.

#### 3.3.6. Correlation Analysis of Key Aroma Compounds

To further investigate the relationships among key aroma compounds, 26 compounds were selected based on the criterion that their concentrations exceeded the odor threshold in more than half of the samples. Pearson correlation analysis was then employed to examine the correlations among these compounds (Figure 3D). Ethyl pentanoate and 1-pentene-3-ol showed a highly significant positive correlation (*r* = 0.998, *p* < 0.01). This relationship may be attributed to their involvement in lipid metabolism and their shared upstream precursor, linolenic acid, which may lead to similar variation trends in the samples. Ethyl hexanoate and hexanal also exhibited a significant positive correlation at the 0.01 level (*r* = 0.986). Hexanal is a typical oxidation product of linoleic acid and can be further oxidized to hexanoic acid, which participates in esterification reactions to form ethyl hexanoate. Therefore, their synchronous variation may reflect a potential continuous transformation relationship of “aldehyde–acid–ester” within the lipid oxidation chain. Moreover, β-pinene, L-limonene, and linalool also displayed significant positive correlations with each other. These compounds belong to the monoterpene class and may share a common biosynthetic precursor, geranyl pyrophosphate. Accordingly, their coordinated accumulation patterns may be associated with similarities in plant-derived terpene biosynthetic pathways.

### 3.4. Microbial Community of SFSP After Storage

#### 3.4.1. Bacterial Species

**Figure 5:**
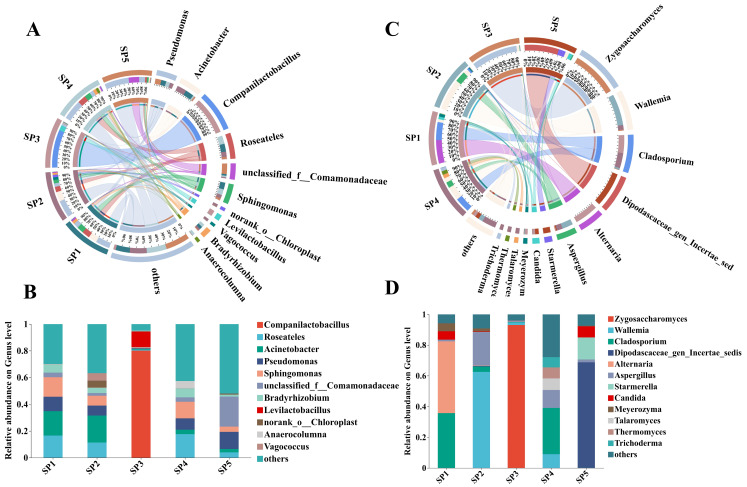
Microbial community of stored SFSP at genus level: bacterial (**A**,**B**) and fungal (**C**,**D**).

To further evaluate the storage safety of SFSP, the microbial community structure and potential microbial risks were analyzed after one month of storage. Based on the genus-level relative abundance analysis (Figure 5B), the bacterial community structures of SFSP samples were generally similar, with communities mainly composed of taxa affiliated with the phyla Firmicutes and Proteobacteria. The Circos plot further illustrates the associations between samples and dominant bacterial genera (Figure 5A), indicating that these core genera were consistently distributed across different samples and constituted the shared bacterial framework of SFSP after storage.

Among the dominant genera, *Companilactobacillus* and *Levilactobacillus*, both belonging to lactic acid bacteria within Firmicutes, showed relatively higher abundances in sample SP3 (Figure 5B). As typical lactic acid bacteria, these genera possess strong acid-producing capabilities and tolerance to low pH environments. Concurrently, lactic acid bacteria play a significant role in enhancing food flavor, texture, and aroma, as well as improving preservation efficacy. Moreover, *Vagococcus* was detected in all samples (Figure 5B). These taxa are generally considered to possess auxiliary metabolic functions and may participate in carbon transformation and intermediate metabolic processes, thereby supporting the continuous generation of flavor precursors. *Vagococcus*, frequently reported in fermented aquatic products and fish sauce, has been associated with amino acid metabolism and ester precursor formation [54]. These microorganisms represent the dominant microbiota in raw fermented sour shrimp paste, playing a crucial role in shaping its distinct flavor profile [1,4]. They are also frequently employed as functional microorganisms in various traditional Asian fermented foods [55]. The persistence of these fermentation-associated bacteria during storage suggests that certain microorganisms may remain viable or re-establish themselves during the post-processing storage period.

In addition to lactic acid bacteria, several genera affiliated with Proteobacteria and closely associated with protein and lipid degradation were commonly detected across the samples (Figure 5B). As common foodborne spoilage bacteria, *Pseudomonas* and *Acinetobacter* species secrete extracellular enzymes that degrade lipids and proteins in meat, producing small-molecule metabolites such as aldehydes, organic acids and ketones, thereby affecting flavor [56]. *Sphingomonas*, meanwhile, is frequently associated with ester metabolism [57].

In summary, fermentation-related bacteria such as *Vagococcus* and *Levilactobacillus* were still detectable in SFSP after one month of storage, while *Pseudomonas* and *Acinetobacter* were identified as the dominant spoilage bacteria within one month of storage. These findings provide a reference for the microbial quality control of SFSP products.

#### 3.4.2. Fungal Species

Most of the fungal genera detected in the samples belonged to stress-tolerant taxa within the phylum *Ascomycota* that are well adapted to acidic environments and thermal processing conditions (Figure 5C), which is consistent with previous reports on acidic fermented aquatic products [58]. Specifically, the fungal community of stir-fried shrimp paste was mainly composed of a limited number of yeast genera, including *Zygosaccharomyces*, *Starmerella*, *Candida*, and *Meyerozyma* (Figure 5D). These yeasts generally exhibit strong tolerance to acidic, saline, and thermal stresses, allowing them to persist under conditions of high salinity, low pH, and heat treatment during processing [59]. In addition, *Wallemia*, *Cryptococcus*, and *Rhodosporidium* were also detected. Among them, *Wallemia* is commonly associated with high-osmotic-pressure food systems and is frequently reported in cured, sugared, and dried products due to its strong environmental adaptability [60]. Its presence reflects the selective pressure exerted by the high osmotic conditions and complex processing procedures involved in stir-fried shrimp paste production.

Overall, except for a few osmophilic fungi, the dominant fungal genera in SFSP after storage were primarily yeasts belonging to the phylum *Ascomycota*. These taxa are generally involved in the formation of flavor precursors through alcoholic fermentation and related enzymatic reactions, thereby contributing to the development of characteristic flavor profiles [61]. Their persistence during storage further indicates a strong capacity to withstand the acidic, saline, and heat-processed conditions characteristic of SFSP.

## 4. Conclusions

This study provides a comprehensive analysis of the flavor characteristics, volatile profiles, and post-storage microbial community of SFSP. Sensory evaluation indicated that SFSP exhibited a well-balanced flavor, retaining the fruity and soy sauce notes of raw sour shrimp paste while also displaying spicy and salty characteristics. Based on HS-SPME-GC-MS analysis, a total of 68 volatile compounds were detected in SFSP, with esters representing the most abundant and with total concentrations varying from 11.48 to 412.94 μg/g (9.58–42.86%), playing a dominant role in the overall aroma profile. Among these volatiles, 26 compounds were identified as aroma contributors. Specifically, ethyl butyrate, ethyl acetate, and ethyl 2-methylbutyrate were responsible for the characteristic fruity notes. Acids such as butanoic acid, acetic acid, and 2-methylbutanoic acid were present at levels well above their odor thresholds, contributing a sour and spicy aroma. Collectively, esters and acids were the most abundant and impactful volatiles, jointly shaping the fruity and sour aroma attributes of SFSP. Microbial community analysis showed that Firmicutes and Proteobacteria were the dominant bacterial phyla, while *Ascomycota* was the prevailing fungal phylum. In addition, the results also revealed certain differences among the samples. Specifically, SP5 exhibited the lowest number of volatile compounds compared with the other four samples, while SP3 showed relatively higher abundances of *Companilactobacillus* and *Zygosaccharomyces* in terms of microbial composition. These differences, to some extent, reflect the insufficient standardization in the traditional fermentation process of sour shrimp paste. Based on the integrated analysis of multi-dimensional data, SP2 better represents the characteristic flavor profile of SFSP, featuring prominent fruity and sour, along with relatively weak fishy and stink attributes, thereby providing a more comprehensive representation of the overall flavor profile of SFSP.

Overall, the results showed that SFSP exhibited relatively consistent common characteristics in terms of sensory attributes, volatile flavor compounds, and microbial composition during storage. In addition, this study provides fundamental data on the flavor formation mechanisms of SFSP in practical processing applications, and also offers insights for microbial safety assessment and risk management. However, this work primarily focused on the characterization of the final stir-fried products and did not involve the fermentation process of the corresponding raw materials or its dynamic changes. On this basis, future studies could expand the sample size and number of batches, and integrate dynamic fermentation monitoring with multi-omics approaches to further elucidate the mechanisms by which raw material characteristics, microbial community succession, and fermentation conditions influence the flavor formation and microbiological safety of the stir-fried product. Such efforts would contribute to a more systematic scientific foundation for the standardization and quality control of traditional fermented condiments.

## Figures and Tables

**Table 1:** The attributes and compositions of the reference standards used in the sensory descriptive analyses of the SFSP.

Attribute	Reference Standard
Fishy	(Freshwater Fish) Fresh gills and ventral membrane from grass carp were finely minced and used as the reference.
	(Sea Fish) Gills and abdominal membrane from pompano were chopped and served as the marine fish reference sample.
Salty	(Cured Meat) Finely diced pieces of traditional Chinese Jinhua ham were used to represent the cured meat salty attribute.
	(Seaweed) Small pieces of dried kelp (kombu) were prepared as the seaweed salty reference.
Stink	(Stinky Tofu) Fermented stinky tofu was crushed into a paste to represent the typical pungent fermented odor.
	(Canned Fermented Fish) Fermented herring from canned surströmming was homogenized into a paste to serve as the reference.
Sour	(Fermented Vegetable) A small amount of pickling brine from Sichuan paocai was used to represent fermented vegetable sourness.
	(Citrus) Fresh lemon pulp (without peel) was crushed and diluted in odor-free water to provide the citrus sour reference.
Fruity	(Apple/Pear) Fresh apple and pear pulp were finely diced and mixed immediately before sensory evaluation.
	(Banana) Ripe banana pulp was cut into small pieces and served as the fruity reference.
	(Strawberry) Fresh strawberry flesh was finely chopped and presented immediately before evaluation.
Spicy	(Pungent Chili) Ground dried chili powder was infused in hot odor-free water and filtered before use.
	(Aromatic Spices) A mixed spice sample containing star anise, Sichuan peppercorn, and cassia cinnamon (equal proportions) was steeped in hot odor-free water and filtered to obtain the aromatic spice reference.
Soy sauce	(Dark Soy Sauce) A small volume of dark soy sauce was used directly as the soy sauce reference.
	(Soybean Paste) Korean doenjang and Chinese Pixian doubanjiang were mixed to represent the fermented soybean paste note.

**Table 2:** pH and total volatile basic nitrogen contents determined in SFSP.

Samples	pH	TVB-N (mg/100 g)
SP1	4.69 ± 0.00 ^c^	90.64 ± 2.12 ^b^
SP2	4.82 ± 0.00 ^b^	96.80 ± 2.27 ^a^
SP3	3.76 ± 0.04 ^d^	66.43 ± 1.54 ^c^
SP4	7.28 ± 0.08 ^a^	52.26 ± 1.25 ^d^
SP5	4.69 ± 0.02 ^c^	13.21 ± 0.31 ^e^

Note: values were expressed as means ± standard deviation (pH: *n* = 2; TVB-N: *n* = 3). Different letters within the same column indicate significant differences (*p* < 0.05). TVB-N: total volatile basic nitrogen.

**Table 3:** Volatile compounds in SFSP samples detected by HS-SPME-GC-MS.

Volatile Compounds	RI ^a^	CAS	Identification ^b^	Threshold ^c^ (μg/kg)	Aroma Description ^d^	Concentration (μg/g) ^e^				
						SP1	SP2	SP3	SP4	SP5
Esters										
Ethyl isobutyrate	973	97-62-1	RI, MS	15 [31]	Fruity, sweet, alcoholic	0.36 ± 0.08 ^b^	1.9 ± 0.33 ^b^	0.6 ± 0.04 ^b^	8.53 ± 1.57 ^a^	nd
Methyl butanoate	991	623-42-7	RI, MS	59 [32]	Fruity, apple, sweet, banana, pineapple	nd	nd	nd	1.12 ± 0.13	nd
Ethyl butanoate	1042	105-54-4	RI, MS	20 [31]	Banana, pineapple, strawberry	3.2 ± 0.22 ^b^	20.56 ± 1.26 ^b^	9.53 ± 0.26 ^b^	188.21 ± 41.96 ^a^	1.59 ± 0.1 ^b^
Ethyl 2-methylbutanoate	1055	7452-79-1	RI, MS	1 [31]	Fruity, citrus	1.64 ± 0.29 ^c^	11.8 ± 3.41 ^ab^	3.4 ± 0.09 ^bc^	18.78 ± 6.8 ^a^	0.75 ± 0.21 ^c^
Ethyl 3-methylbutanoate	1070	108-64-5	RI, MS	3 [31]	Fruity, apple, banana	nd	nd	0.95 ± 0.01 ^b^	5.37 ± 1.06 ^a^	nd
Ethyl pentanoate	1139	539-82-2	RI, MS	94 [32]	Sweet, fruity, apple, pineapple, green	0.66 ± 0.17 ^b^	6.88 ± 0.93 ^a^	1.42 ± 0.16 ^b^	7.31 ± 0.95 ^a^	nd
Methyl 4-methylpentanoate	1147	2412-80-8	RI, MS		Fruity, sweet, banana, pineapple, cheese	nd	0.32 ± 0	nd	nd	nd
Isobutyl butyrate	1162	539-90-2	RI, MS	75 [33]	Sweet, fruity, pineapple, rum, cherry, apple	0.7 ± 0.04 ^b^	2.39 ± 0.08 ^a^	nd	nd	nd
Ethyl isohexanoate	1198	25415-67-2	RI, MS		Fruity	32.13 ± 0.86 ^b^	135.14 ± 15.78 ^a^	4.54 ± 0.51 ^c^	8.65 ± 2.33 ^c^	nd
Butyl butanoate	1221	109-21-7	RI, MS	400 [32]	Fruity, banana, pineapple, green, cherry	0.2 ± 0.14 ^b^	0.76 ± 0.29 ^a^	nd	nd	nd
Ethyl hexanoate	1236	123-66-0	RI, MS	14 [34]	Banana, green apple	0.31 ± 0.12 ^c^	1.89 ± 0.06 ^c^	5.77 ± 0.32 ^b^	18.8 ± 1.85 ^a^	nd
Ethyl (E)-3-hexenoate	1294	26553-46-8	RI, MS		Sweet, green, fruity, passion fruit, pineapple	1.63 ± 0.05 ^b^	5.72 ± 0.32 ^a^	nd	nd	nd
Ethyl heptanoate	1336	106-30-9	RI, MS	300 [35]	Fruity	nd	0.31 ± 0.06 ^c^	0.45 ± 0.08 ^b^	1.64 ± 0.04 ^a^	nd
Ethyl octanoate	1437	106-32-1	RI, MS	580 [36]	Sweet, floral, fruity, banana, pear	nd	nd	0.55 ± 0.03 ^b^	1.47 ± 0.22 ^a^	nd
Linalyl formate	1560	115-99-1	RI, MS		Citrus, herbal, soapy, fatty, green, woody	2.44 ± 0.09 ^b^	9.67 ± 0.63 ^a^	nd	nd	nd
Linalyl acetate	1560	115-95-7	RI, MS		Sweet, green, citrus, bergamot, lavender, woody	7.16 ± 0.26 ^b^	26.63 ± 2.16 ^a^	nd	nd	nd
Ethyl 3-(methylthio)propionate	1574	13327-56-5	RI, MS	8.45 [32]	Sulfury, metallic, pineapple, fruity, tomato	0.34 ± 0.22 ^b^	nd	0.39 ± 0.08 ^b^	1.57 ± 0.73 ^a^	nd
Ethyl decanoate	1642	110-38-3	RI, MS	200 [34]	Fruity, fatty, pleasant	nd	nd	0.06 ± 0	nd	nd
Diethyl succinate	1676	123-25-1	RI, MS		Fruity, cooked apple	0.09 ± 0.05 ^c^	nd	1.07 ± 0.1 ^b^	3.28 ± 0.75 ^a^	nd
Ethyl benzeneacetate	1794	101-97-3	RI, MS	575 [34]	Fruity	3 ± 0.09 ^c^	6.71 ± 0.57 ^c^	18.12 ± 1.81 ^b^	34.67 ± 3.77 ^a^	6.21 ± 0.25 ^c^
β-Phenethyl acetate	1826	103-45-7	RI, MS	1800 [36]	Fruity, rose	nd	0.41 ± 0.11 ^a^	0.06 ± 0.01 ^b^	nd	nd
Methyl β-phenylpropionate	1858	103-25-3	RI, MS		Honey, fruity, wine, balsam, floral	0.62 ± 0.2 ^b^	2.59 ± 0.07 ^a^	0.27 ± 0.2 ^c^	nd	nd
Ethyl 3-phenylpropanoate	1900	2021-28-5	RI, MS	1.6 [34]	Hyacinth, rose, honey, fruity, rum	59.98 ± 1.73 ^b^	174.64 ± 8.63 ^a^	16.2 ± 0.82 ^d^	31.88 ± 5.18 ^c^	2.64 ± 0.18 ^e^
2-Phenylethyl butanoate	1972	103-52-6	RI, MS		Musty, sweet, floral, yeast, strawberry	0.14 ± 0.07 ^a^	0.25 ± 0.1 ^a^	nd	nd	nd
Ethyl myristate	2042	124-06-1	RI, MS	2000 [37]	Waxy, soap	0.38 ± 0.12 ^a^	0.32 ± 0.04 ^b^	0.09 ± 0.04 ^c^	nd	nd
Ethyl hexadecanoate	2221	628-97-7	RI, MS	1000 [35]	Mild waxy	0.62 ± 0.08 ^a^	0.81 ± 0.28 ^a^	0.38 ± 0.1 ^a^	0.56 ± 0.37 ^a^	0.29 ± 0.1 ^a^
Ethyl Acetate	907	141-78-6	RI, MS	5 [32]	Fruity, pineapple, sweet	2.27 ± 0.12 ^b^	2.66 ± 0.73 ^b^	2.49 ± 0 ^b^	31.57 ± 1.56 ^a^	nd
Ethyl propanoate	965	105-37-3	RI, MS	100 [32]	Sweet, fruity, rum, grape, pineapple	0.48 ± 0.2 ^b^	0.58 ± 0.24 ^b^	0.55 ± 0.06 ^b^	6.62 ± 0.85 ^a^	nd
Ketones										
3-Hexanone	1051	589-38-8	RI, MS	41 [32]	Sweet, fruity, rum, grape	3.75 ± 0.05 ^b^	25.2 ± 1.01 ^b^	5.9 ± 8.34 ^b^	194.16 ± 29.72 ^a^	2.24 ± 0.29 ^b^
2,5-Dimethyl-3-hexanone	1135	1888-57-9	RI, MS			0.51 ± 0.25 ^b^	6.03 ± 1.13 ^a^	1.07 ± 0.06 ^b^	6.03 ± 0.88 ^a^	0.21 ± 0.04 ^b^
2,4-Dimethyl-3-hexanone	1170	18641-70-8	RI, MS	1900 [32]		0.23 ± 0.12 ^a^	nd	0.32 ± 0 ^a^	0.33 ± 0.01 ^a^	nd
Terpenoids										
Camphene	1062	79-92-5	RI, MS	1860 [32]	Woody, herbal, fir needle, camphor	0.92 ± 0.04 ^b^	3.52 ± 0.02 ^a^	0.55 ± 0.1 ^c^	nd	nd
Sabinen	1125	3387-41-5	RI, MS		Woody, terpene, citrus, pine, spice	2.37 ± 0.13 ^b^	23.57 ± 0.93 ^a^	0.43 ± 0.1 ^c^	0.3 ± 0.07 ^c^	nd
β-Myrcene	1130	123-35-3	RI, MS		Peppery, terpene, spicy, balsam	1.62 ± 0.13 ^c^	13.59 ± 1.23 ^a^	0.24 ± 0.06 ^c^	7.47 ± 2.11 ^b^	nd
β-Pinene	1167	127-91-3	RI, MS	140 [32]	Dry, woody, resinous, pine, hay, green	4.03 ± 0.36 ^b^	20.21 ± 1.1 ^a^	0.43 ± 0.01 ^c^	nd	nd
L-Limonene	1203	5989-54-8	RI, MS	38 [33]	Terpene, pine, herbal, peppery	7.72 ± 0.23 ^b^	30.55 ± 2.96 ^a^	1.72 ± 0.23 ^c^	2.77 ± 0.47 ^c^	0.3 ± 0.01 ^c^
Eucalyptol	1213	470-82-6	RI, MS		Eucalyptus, herbal, camphor	2.82 ± 0.11 ^c^	11.71 ± 1.12 ^a^	5.48 ± 0.22 ^b^	5.34 ± 0.68 ^b^	nd
Linalool	1542	78-70-6	RI, MS	15 [31]	Citrus, floral, sweet, woody, green	10.77 ± 0.99 ^b^	62.35 ± 4.18 ^a^	0.54 ± 0.1 ^c^	1.03 ± 0.2 ^c^	0.56 ± 0 ^c^
Terpinen-4-ol	1611	562-74-3	RI, MS	110 [38]	Pepper, woody, musty, sweet	0.95 ± 0.06 ^b^	1.5 ± 0.12 ^a^	1.26 ± 0.06 ^a^	1.37 ± 0.19 ^a^	nd
α-Curcumene	1786	644-30-4	RI, MS		Herbal	1.06 ± 0.07 ^b^	3.18 ± 0.31 ^a^	nd	nd	nd
Acids										
Acetic acid	1441	64-19-7	RI, MS	22,000 [32]	Pungent, sour, vinegar	20.64 ± 3.51 ^b^	51.33 ± 0.39 ^a^	37.11 ± 5.77 ^ab^	34.71 ± 12.97 ^ab^	22.88 ± 1.83 ^b^
Propanoic acid	1528	79-09-4	RI, MS	5.7 [33]	Pungent, acidic, cheesy, vinegar	3.05 ± 0.7 ^bc^	11.73 ± 1.32 ^a^	3.55 ± 0.63 ^b^	2.8 ± 0.26 ^bc^	1.19 ± 0.16 ^c^
Isobutanoic acid	1558	79-31-2	RI, MS		Acidic, sour, cheese, dairy, buttery, rancid	16.02 ± 0.79 ^b^	53.06 ± 3.15 ^a^	2.08 ± 0.4 ^d^	8.76 ± 1.38 ^c^	2.84 ± 0.32 ^d^
Butanoic acid	1618	107-92-6	RI, MS	2500 [35]	Cheese, rancid	98.74 ± 6.38 ^c^	219.4 ± 13.14 ^a^	39.99 ± 10.11 ^d^	137.89 ± 19.07 ^b^	46.5 ± 1.94 ^d^
2-Methylbutanoic acid	1661	116-53-0	RI, MS	2200 [32]	Pungent, acid, roquefort cheese	48.68 ± 0.35 ^b^	127.39 ± 7.08 ^a^	15.07 ± 2.89 ^c^	45.95 ± 8.31 ^b^	13.5 ± 0.57 ^c^
Pentanoic acid	1728	109-52-4	RI, MS	0.037 [33]	Sickening, putrid, acidic, sweaty, rancid	2.29 ± 0.04 ^c^	10.97 ± 0.69 ^a^	1.11 ± 0.15 ^d^	3.18 ± 0.22 ^b^	0.17 ± 0.02 ^e^
Isobutylacetic acid	1792	646-07-1	RI, MS	0.4 [33]	Spicy, cheese	24.62 ± 0.81 ^b^	66.8 ± 7.38 ^a^	0.69 ± 0.06 ^c^	1.24 ± 0.26 ^c^	nd
Aldehydes										
Hexanal	1072	66-25-1	RI, MS	5 [32]	Green grass, fruity, sweaty	0.18 ± 0.05 ^b^	1.32 ± 0.51 ^b^	1.61 ± 0.34 ^b^	10.14 ± 1.79 ^a^	nd
3-Methylpentanal	1044	15877-57-3	RI, MS	0.81 [32]		0.69 ± 0.07 ^c^	4.6 ± 0.79 ^b^	1.28 ± 0.17 ^c^	8.36 ± 2.06 ^a^	0.16 ± 0 ^c^
2-Methylbutanal	926	96-17-3	RI, MS	30 [33]	Musty, coffee, nutty, fatty, alcoholic	1.37 ± 0.31 ^a^	nd	nd	nd	1.32 ± 0.25 ^a^
Other										
Toluene	1062	108-88-3	RI, MS	527 [32]	sweet	nd	nd	nd	nd	0.34 ± 0.13
p-Xylene	1125	106-42-3	RI, MS	58 [33]	phenolic	nd	nd	nd	nd	1.93 ± 0.03
Benzaldehyde	1125	100-52-7	RI, MS	2000 [36]	Roasted, almond	0.16 ± 0.05 ^c^	nd	0.81 ± 0.04 ^b^	1.84 ± 0.34 ^a^	0.35 ± 0.02 ^c^
3-Methylbutyramide	1130	541-46-8	RI, MS			nd	nd	0.07 ± 0.09	nd	nd
2-Acetylpyrrole	1167	1072-83-9	RI, MS	170,000 [32]	Musty, licorice, walnut, bready	1.49 ± 0.08 ^b^	1.85 ± 0.45 ^ab^	nd	nd	2.28 ± 0.13 ^a^
Phenol	1203	108-95-2	RI, MS	5900 [39]	Phenolic, plastic, rubber	4.27 ± 0.09 ^b^	39.76 ± 4.49 ^a^	3.17 ± 0.3 ^b^	5.27 ± 0.7 ^b^	4.02 ± 0.12 ^b^
Indole	1205	120-72-9	RI, MS	40 [32]	Animal, floral, mothball	0.79 ± 0.5 ^b^	2.95 ± 0.36 ^a^	nd	nd	nd
Sulfides										
2,3-Dithiabutane	1077	624-92-0	RI, MS	2.2 [33]	Vegetables, cabbage, onion	0.23 ± 0.18 ^b^	0.87 ± 0.28 ^a^	nd	nd	nd
Methyl trisulfide	1394	3658-80-8	RI, MS	4 [33]	Cooked, onion, savory, meaty	1.33 ± 0.02 ^b^	2.88 ± 0.51 ^a^	nd	nd	nd
Diallyl disulphide	1491	2179-57-9	RI, MS	0.22 [33]	Alliaceous, onion, garlic	6.28 ± 0.31 ^b^	31.58 ± 2.38 ^a^	2.46 ± 0.01 ^c^	nd	nd
Furans										
2-Furanmethanol	1655	98-00-0	RI, MS	2000 [32]	Alcoholic, musty, sweet, caramel, bread, coffee	0.58 ± 0.1 ^b^	1.89 ± 0.86 ^a^	0.22 ± 0.03 ^b^	1.18 ± 0.27 ^ab^	2.05 ± 0.11 ^a^
2,5-Dimethyltetrahydrofuran	853	1003-38-9	RI, MS			nd	nd	nd	nd	0.68 ± 0
Alcohols										
Isobutanol	1087	78-83-1	RI, MS	40,000 [31]	Solvent, bitter, chemical	nd	nd	nd	nd	0.73 ± 0.04
1-Butanol	1135	71-36-3	RI, MS	150,000 [36]	Medicinal, phenolic	nd	2.27 ± 0.15 ^b^	0.54 ± 0.02 ^c^	2.94 ± 0.03 ^a^	nd
1-Pentene-3-ol	1126	616-25-1	RI, MS	400 [32]	Green, radish, vegetable, fruity	0.51 ± 0.25 ^b^	6.03 ± 1.13 ^a^	1.07 ± 0.06 ^b^	6.03 ± 0.88 ^a^	0.21 ± 0.04 ^b^
2-Methyl-4-butanol	1202	123-51-3	RI, MS	50,000 [36]	Almond, toasted	nd	nd	0.41 ± 0.06 ^b^	0.97 ± 0.2 ^a^	0.46 ± 0.01 ^b^
Phenethyl alcohol	1915	60-12-8	RI, MS	14,000 [34]	Rose, honey	7.16 ± 0.28 ^b^	17.58 ± 0.84 ^a^	3.47 ± 0.41 ^c^	3.28 ± 0.07 ^c^	3.47 ± 0.71 ^c^
Pyridines										
Tetramethylpyrazine	1479	1124-11-4	RI, MS	1000 [32]	Nutty, musty, chocolate, coffee, cocoa, burnt	nd	nd	0.07 ± 0.04	nd	nd

^a^ RI, retention index calculated from n-alkanes (C6–C24) under identical chromatographic conditions. ^b^ Identification was based on mass spectrum (MS) comparison with the NIST mass spectral library (minimum similarity score of 80%), retention index (RI) agreement with reference values from the NIST database (maximum permitted deviation (ΔRI) of 50 index units). ^c^ References: [31] (Guth, 1997); [32] (Van Gemert, 2011); [36] (Peinado et al., 2004) [33] https://www.chemicalbook.com; [34] (Ferreira et al, 2000); [35] (Moyano et al., 2002); [37] (Tao & Zhang, 2010); [38] (Oliveira et al., 2004); [39] (Maga & Katz, 1978) ^d^ Aroma descriptions of the compounds were obtained from https://thegoodscentscompany.com/search.php (accessed on 15 November 2025). ^e^ Concentration data were presented as mean ± standard deviation (*n* = 2). nd: not detected. Values in the same row with different letters (a–e) indicate statistically significant differences between groups (*p* < 0.05).

## Data Availability

The original contributions presented in the study are included in the article/Supplementary Material, further inquiries can be directed to the corresponding authors.

## Supplementary Materials

The following supporting information can be downloaded at: https://www.mdpi.com/article/10.3390/foods15132338/s1, Table S1. Mean sensory scores (mean ± SD) and statistical comparisons of SFSP samples.
