# Supplementary material for: A Comprehensive Study on the Volatile Flavor Profile and Microbial Community of Stir-Fried Sour Shrimp Paste
Source: Foods. 2026 Jul 1;15(13):2338. doi: 10.3390/foods15132338 (PMC13361481; doi:10.3390/foods15132338)
Supplement: Supplementary file 1 [file foods-15-02338-s001.zip › foods-4383401-supplementary.pdf]

SUPPLEMENTARY INFORMATION FOR

A comprehensive study on the volatile flavor profile and microbial community of stir-fried sour shrimp paste

Jiahui Shi[1,2,3,#], Weixi Yang[1,2,3,#], Huangqing Yang[1,2,3], Kangli Guo[1,2,3], Wenlu Li[1,2,3], Yuxiang Gu[1,2,3*] and Yanbo Wang[1,2,3*]

[1] School of Food and Health, Beijing Technology and Business University, Beijing 100048, China

[2] Key Laboratory of Geriatric Nutrition and Health, Ministry of Education, Beijing Technology and Business University, Beijing 100048, China

[3] cNHC Specialty Laboratory of Food Safety Risk Assessment and Standard Development, Beijing Technology and Business University, Beijing 100048, China

[#]These authors contributed equally to this work.

*Corresponding author at: School of Food and Health, Beijing Technology and Business University, Beijing 100048, China

E-mail address: yuxianggu@btbu.edu.cn. (Yuxiang Gu),wyb1225@163.com (Yanbo Wang)

Table S1 Mean sensory scores (mean ± SD) and statistical comparisons of SFSP samples

	SP1	SP2	SP3	SP4	SP5
Spicy	$4.5±2.13^{a}$	$4.95±1.77^{a}$	$4±0.89^{a}$	$4.25±2.47^{a}$	$4.7±1.93^{a}$
Fruity	$3.9±2.39^{a}$	$4.25±2.95^{a}$	$3.75±2.23^{a}$	$4.15±1.96^{a}$	$4.05±1.85^{a}$
Soy sauce	$4.2±1.49^{a}$	$3.25±1.64^{a}$	$3.85±2.03^{ab}$	$3.7±1.83^{ab}$	$2.8±1.81^{b}$
Salty	$3.45±2.72^{a}$	$3.85±2.38^{a}$	$3.05±1.41^{a}$	$4.1±2.39^{a}$	$2.9±2.45^{a}$
Fishy	$3.25±1.81^{a}$	$2.7±1.51^{a}$	$3.35±1.41^{a}$	$3.5±1.06^{a}$	$2.6±1.6^{a}$
Stink	$2.6±1.06^{a}$	$2.65±0.89^{a}$	$2.55±1.13^{a}$	$2.85±0.83^{a}$	$3.55±1.91^{a}$
Sour	$2.5±1.75^{a}$	$2.95±2.47^{a}$	$2.45±1.89^{a}$	$2.25±2.03^{a}$	$2.5±1.2^{a}$

Note: Values are presented as mean ± standard deviation of sensory scores obtained from 20 trained panelists ($n = 20$). Different letters within the same row indicate significant differences ($p < 0.05$).
